# Preschool Hearing Screening: Nineteen Years of the Coração Delta Project in Campo Maior, Portugal

**DOI:** 10.3390/audiolres15040104

**Published:** 2025-08-11

**Authors:** Cláudia Reis, Luísa Monteiro, Conceição Monteiro, Joana Pereira, Joana Teixeira, João Mendes, Mariana Pereira, Magda Barrocas, Dionísia Gomes, Margarida Serrano

**Affiliations:** 1 Polytechnic University of Coimbra, Rua da Misericórdia, Lagar dos Cortiços, S. Martinho do Bispo, 3045-093 Coimbra, Portugal; creis@estesc.ipc.pt (C.R.);; 2 Hospital Lusíadas, Rua João Freitas Branco, 1500-458 Lisboa, Portugal; luisamonteiro.ent@gmail.com (L.M.);; 3 Associação Coração Delta, 7370-050 Campo Maior, Portugal; magda.barrocas@delta-cafes.pt (M.B.); dionisia.gomes@gruponabeiro.com (D.G.); 4 H&TRC-Health & Technology Research Center, Polytechnic University of Coimbra, Rua da Misericórdia, Lagar dos Cortiços, S. Martinho do Bispo, 3045-093 Coimbra, Portugal

**Keywords:** hearing screening, tympanogram, children, preschool

## Abstract

**Background/Objectives**: Preschool hearing screening is justified by the risk of late onset hearing loss, the high prevalence of otitis media with effusion in school-aged children, and the critical timing just before children begin formal reading and learn to write. This study describes the results of the annual preschool hearing screening program in Campo Maior from 2007 to 2025 (nineteen years) and correlates the audiological referral to the otoscopy findings by the otolaryngologists. **Methodology**: Retrospective study using clinical records from nineteen years of preschool hearing screening. **Results**: Screening identified 310 children (29% of 1068 screened) requiring referral to an ENT specialist. Of the 217 referred children evaluated by ENT, 198 (91.2%) had confirmed pathology or healthcare needs of medical intervention. A statistically significant positive association (r = 0.254, *p* < 0.05) existed between abnormal otoscopy findings and Type B or C2 tympanograms (versus Type A or C1). Hearing loss occurring with Type A tympanograms (0.8% unilaterally, 0.3% bilaterally) may suggest sensorineural hearing loss. **Conclusion**: This study reinforces the importance of universal preschool audiological screening for all children, particularly for children facing geographic barriers to healthcare. Community-based interventions facilitated by social solidarity associations can play a crucial role in mitigating healthcare access disparities across populations.

## 1. Introduction

Children develop literacy through progressive phases (pre-alphabetic, partial alphabetic, alphabetic, and consolidated alphabetic), moving beyond oral language to acquire written language concepts, attentive listening skills, and exposure to richer vocabulary and syntax, facilitated by sharing ideas [1,2,3]. This process fundamentally requires learning the relationship between orthography and phonemes [4,5], with hearing quality being critically important, especially during the partial and alphabetic phases. During these phases, children must develop phonemic awareness to detect individual speech sounds and acquire grapheme-phoneme correspondence knowledge for decoding, linking written words to their stored auditory lexicon [4,6]. As children spend significant classroom time listening to learn—including learning to read—acoustic conditions are paramount for clear speech perception. Children are particularly vulnerable to adverse acoustics (e.g., background noise masking word boundaries) because their auditory systems are immature until ages 12–14, they are continuously learning new sounds and concepts requiring redundancy, and they need lower hearing thresholds (below 16 dB) than adults for effective perception of low-intensity, rapid speech sounds [7,8,9,10,11,12]. Consequently, both the student’s hearing and the classroom’s acoustics directly impact the clarity of information essential for learning [7,8]. To prevent cognitive-linguistic delays and ensure timely entry into the alphabetic phase, auditory issues must be identified early.

Early detection and prevention of hearing loss are essential for mitigating its long-term impact on academic achievement and socio-professional inclusion. These efforts must extend beyond the neonatal period and encompass the preschool and early school years [13]. Although universal newborn hearing screening is well implemented in Portugal, the prevalence of hearing loss nearly doubles by preschool age. This increase is largely attributed to conditions such as otitis media with effusion (OME) and late-onset permanent hearing loss [13]. Hearing loss at approximately five years of age—whether conductive (often due to OME) or sensorineural—frequently remains undiagnosed. This is often due to its presentation as unilateral, fluctuating, or late-onset in nature, caused by genetic, traumatic, or infectious etiologies, and may not noticeably disrupt early speech and language development [14,15]. The high prevalence of OME—affecting up to 90% of children before school entry and typically causing average hearing thresholds of 25–30 dB—combined with the possibility of late-onset permanent loss and the proximity to the start of formal learning of reading and writing justify the implementation of preschool screening [15,16,17,18].

Preschool hearing screening targets all children aged 4 to 7 years without a prior diagnosis of hearing impairment, with the dual objective of enabling early and reliable detection of hearing loss and promoting equitable access to care, regardless of parental knowledge or socioeconomic status [14,15,19]. To minimize the risk of missed cases (false negatives), the protocol emphasizes sensitivity over specificity, accepting a higher rate of false positives (over-referrals) [15]. The screening procedure typically includes three brief, child-appropriate assessments [13,14,20]: otoscopy, which checks for the presence or absence of cerumen or any impossibility of performing tympanometry and/or hearing screening (e.g., transtympanic tube) [14]; tympanogram, which indirectly assesses the compliance of the tympanic membrane and the pressure within the middle ear. Its sensitivity (ability to detect middle ears with effusion—type B tympanogram), according to studies, ranges from 49% to 99%, while its specificity (ability to diagnose normal middle ears—type A tympanogram) is approximately 100% in most studies [21]; and hearing screening, using the hear/not-hear criterion at frequencies of 1, 2, and 4 kHz—with the addition of 0.5 kHz when tympanometry is not available and ambient noise conditions allow—to detect both conductive and sensorineural deficits [13,22].

Access to healthcare should be a universal right, and for the World Health Organization, the economic and social circumstances of individuals should not condition their maximum health potential [23]. In Portugal, this principle is constitutionally enshrined: Article 64 of the Constitution guarantees the fundamental right to health protection, mandating adherence to the democratic principles of universality and equality regardless of how care is accessed or financed [24,25]. To uphold this right, services must be provided close to users’ residences to minimize time and travel burdens, particularly in isolated areas. This requires local collaboration between health institutions, professionals, authorities, and the social sector, while ensuring technical standards for effective service delivery [24,25].

Coração Delta, a social solidarity association, exemplifies effective collaboration between the social sector and health institutions/professionals in the inland Portuguese municipality of Campo Maior. Its mission focuses on driving projects in education, special education, health, and social intervention. Campo Maior, a border town in the Alto Alentejo region (District of Portalegre), had a 2023 resident population of 7916, including 1125 children aged 0–14 years. Access to specialized audiology and otolaryngology services in this region is extremely limited, resulting in significant delays for necessary referrals and care [26,27].

Using clinical records spanning nineteen years (2007–2025), this retrospective study aims to describe the results of the annual preschool audiological screening conducted in the Municipality of Campo Maior from 2007 to 2025 (nineteen years) and to correlate the audiological referral to the otoscopy findings by the otolaryngologists.

## 2. Materials and Methods

The audiological screening of the children was conducted, in all instances, in the presence of their legal guardian or a duly authorized representative. Currently, legal guardians sign an informed consent form authorizing the transfer of data to the Coração Delta Association.

This retrospective study using clinical records from nineteen years of preschool hearing screening study was carried out in compliance with established ethical principles and in full accordance with the General Data Protection Regulation (GDPR—Regulation (EU) 2016/679). Anonymity and confidentiality of all participant data were strictly maintained, in line with best practices for research involving human subjects and the Declaration of Helsinki.

### 2.1. Participants

Over a nineteen-year period, a total of 1068 children from the Municipality of Campo Maior were screened prior to entering the first grade of primary school in the upcoming academic year. Among the screened children, 534 were male and 534 were female. Most of the children, specifically 828 (77.5%), were aged five, while the remaining 240 (22.5%) were aged six.

### 2.2. Procedure

Annually, Coração Delta staff contact all preschools in the municipality of Campo Maior to identify children transitioning to the first grade of primary education. After identification, the respective legal guardians are contacted and invited to express their interest in their child’s participation in the screening. Once the interest is confirmed, the legal guardians are informed about the date, time, and location of the screening. To facilitate family participation, the screening has always been scheduled for Saturdays, ensuring greater availability for involvement.

The screening was conducted annually, in March (53.2%), April (27.6%), May (8.2%), or June (11.0%), by a team consisting of two professors and four students from the Audiology Bachelor’s degree program at the Coimbra Health School and two Otolaryngologists (ENT). The evaluations took place in two rooms prepared to ensure minimal ambient noise.

Each year, two otoscopes, two impedance meters, and two audiometers, all properly calibrated, were used.

**Figure 1:**
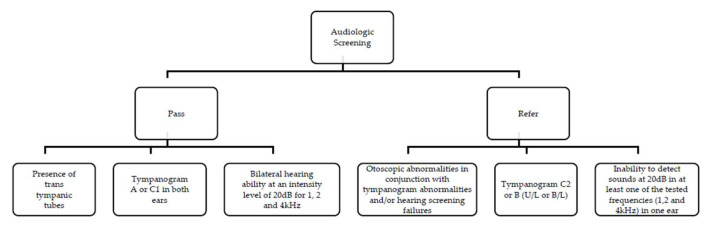
Screening protocol.

Children were screened according to the following protocol: otoscopy, tympanogram, and hearing screening at an intensity of 20 dB in 1, 2 and 4 kHz (Figure 1).

The child successfully passed the screening under the following conditions:

– The presence of transtympanic tubes, accompanied by a recommendation to continue consulting their otorhinolaryngologist (ENT).
– A tympanogram results classified as type A or C1 in both ears.
– Bilateral hearing ability at an intensity level of 20 dB for frequencies of 1, 2, and 4 kHz.

The child was referred for further evaluation based on the following criteria:

– Observed alterations during otoscopy, in conjunction with tympanogram abnormalities and/or hearing screening failures.
– A tympanogram results classified as type C2 or B in one or both ears.
– Inability to detect sounds at 20 dB in at least one of the tested frequencies in one ear.

The referred children were promptly seen by an ENT specialist at the screening site, upon their request.

### 2.3. Statistical Analysis

Descriptive statistics summarized discrete variables as frequencies and percentages. Spearman’s correlation assessed associations between variables, with statistical significance defined as *p* < 0.05.

## 3. Results

### 3.1. Screening Results

Among the 1068 children who were screened, 310 (29%) did not pass the screening and were subsequently referred to an ENT specialist. Of those referred, unilateral changes were observed in 137 children (12.7%), while 173 children exhibited bilateral changes (16.3%). The most common findings were alterations in the tympanogram, occurring unilaterally in 104 children (9.7%) and bilaterally in 81 children (7.6%). 7.6% of the children presented with a type B tympanogram in both ears. Tympanostomy tubes were present in only 12 (1.2%) of the screened children. Hearing loss was associated with the presence of a type B tympanogram, occurring in the right ear in 50.9%, 29.8%, and 39.5% of cases at 1, 2, and 4 kHz, respectively. In the left ear, it occurred in 44.6%, 31.5%, and 38.7% of cases at 1, 2, and 4 kHz, respectively (Table 1, Table 2 and Table 3).

In 2021, due to COVID-19, 87 children from 2020 and 2021 were screened. That year, only 11.5% of the children did not pass the screening. Up until that moment, the referral percentage was 29.5%. After 2021, the referral percentage increased to 34.2%. In 2022, there was a significant increase in referrals, with 38.9% of the children being referred.

### 3.2. Medical Referral

The ENT observation at the time of the screening process was conducted for 217 children, representing 70% of the referrals from the screening, and early treatment was ensured whenever possible (wax removal, toilette of middle ear discharge, prescription of medical treatment). All the children confirmed to have otoscopic or audiological findings were referred to a formal hospital evaluation. Of these, 123 children (56.7%), were directed to a follow-up evaluation by the ENT specialty, while only 19 children (8.8%) did not receive any type of medical referral (Table 4).

### 3.3. Association of Medical Otoscopy Findings with Tympanogram Types

Table 5 shows the relationship between the tympanogram results of 434 ears and the otoscopy performed by an ENT physician. Among the ears examined, 204 were considered normal, and 109 had otitis media with effusion. Additionally, one ear was identified with a perforation and 29 with Eustachian tube dysfunction. Type B and Type C2 tympanograms were the ones with the highest percentage of associated pathology (80.9% for Type B and 46.5% for Type C2).

A statistically significant positive association was observed between abnormal medical otoscopy findings and Type B or C2 tympanograms versus Type A or C1 tympanograms (r = 0.254; *p* < 0.001; N = 434) and between abnormal medical otoscopy findings and Type B tympanograms versus Type A tympanograms (r = 0.566; *p* < 0.001; N = 215).

A statistically significant positive association was also observed between abnormal medical otoscopy findings and Type B versus Type A or C1 or C2 tympanograms (r = 0.456; *p* < 0.001; N = 434).

## 4. Discussion

This retrospective study aims to describe the results of the annual preschool audiological screening conducted in the Municipality of Campo Maior from 2007 to 2025 (nineteen years) and to correlate the audiological referral to the otoscopy findings by the otolaryngologists.

Among 1068 children screened, 310 (29%) failed the screening and were referred for ENT evaluation on site.

Comparable findings were reported in a study assessing the prevalence of hearing loss among school-aged children (8–13 years old) from rural and urban regions of mid-eastern Poland, using standard audiological assessments—pure tone audiometry, tympanometry, and otoacoustic emissions. Results from all three tests were significantly poorer in children from rural areas compared to their urban counterparts: 10.1–23.1% for tympanometry, 3–9.7% for pure tone audiometry, and 17.3–31.8% for otoacoustic emissions. Notably, pure tone audiometry yielded the lowest proportion of not pass results [28].

Subsequent assessment by ENT specialists of 217 referred children confirmed 198 (91.2%) cases with pathology or healthcare needs requiring medical intervention.

Abnormal medical otoscopy findings significantly predict Type B/C2 tympanograms (vs. Type A/C1), with a moderate positive correlation (r = 0.254). This confirms tympanometry’s sensitivity to middle ear effusion and other otoscopically detectable pathologies. These findings are consistent with those reported by Anwar et al. (2016), who observed a tympanogram sensitivity of 85.85% and a specificity of 72.22% in the identification of otitis media with effusion. Similarly, Fiellau-Nikolajsen (1980) reported a sensitivity of 91% and a specificity of 84%, further reinforcing the diagnostic utility of tympanometry in the assessment of middle ear conditions [29,30].

A related study involving Alaskan children from preschool through 12th grade evaluated several hearing screening methods, including pure-tone audiometry, otoacoustic emissions, and tympanometry, with the addition of high-frequency testing. Findings indicated that including tympanometry in the screening protocol increased sensitivity by approximately 20% when combined with pure-tone testing, further confirming its essential role in comprehensive audiological assessments [31].

Regarding hearing loss, it is more frequently associated with type B tympanograms than with type A. In fact, a type B tympanogram is indicative of otitis media with effusion, and the literature reports that this condition can cause mild to moderate hearing loss [17,18].

Conversely, hearing loss associated with a Type A tympanogram (0.8% unilateral and 0.3% bilateral) is likely indicative of sensorineural hearing loss, potentially of mild to moderate severity, which had not yet been detected by the ages of 5 or 6. These findings suggest a higher prevalence of sensorineural hearing loss compared to that reported by Brodie et al. (2022), who identified a prevalence of 0.2% in a screening of children aged 2 to 6 years, and by Lü et al. (2011), who reported a prevalence of 0.75 per 1000 (95% CI: 0.38–1.12) in children aged 3 to 6 years [32,33].

These findings confirm that preschool hearing screening is justified, given the incidence of late-onset hearing loss, the high prevalence of otitis media with effusion, and its critical importance preceding formal reading and writing learning. Most identifiable conditions can be resolved through medical or surgical intervention, which typically restores hearing thresholds to normal levels. Early identification is fundamental for establishing proper foundational literacy skills—the foundation of all lifelong learning.

To enhance the effectiveness of future screening protocols, it is crucial to establish clear criteria for what constitutes a normal otoscopic examination and to determine whether, and under which specific circumstances, referral should be initiated based solely on otoscopic findings. The incorporation of otoacoustic emissions as a standard method in hearing screening protocols should also be considered.

Future research should also examine the longitudinal outcomes of referred children, addressing both medical management—including pharmacological treatment and surgical interventions—and audiological follow-up, particularly in cases with suspected sensorineural hearing loss. The present study’s authors observed that, frequently with financial support from Coração Delta, the majority of referred children received appropriate and timely care at tertiary hospitals, notably in Évora and Lisbon.

This study confirms the importance of preschool screening for all children, especially those from more vulnerable populations, such as those geographically distant from healthcare services. Community intervention promoted by social solidarity associations can play a crucial role in mitigating disparities in access to healthcare services among diverse populations.

Moreover, there is a well-documented need for systematic hearing screening in school-age children, as acquired hearing impairments during this critical developmental period can significantly hinder cognitive function and academic achievement.

## Figures and Tables

**Table 1:** Summary of screening results (N = 1068 children).

	N	%
Children passed screening	758	71.0
Children referred to ENT	310	29.0
–With unilateral changes	137	12.7
–With bilateral changes	173	16.3
Tympanogram alterations		
–Unilateral	104	9.7
–Bilateral	81	7.6
–Type B tympanogram in both ears	81	7.6
Tympanostomy tubes present	12	1.2

N—number; %—percentage.

**Table 2:** Association of type B tympanogram with hearing loss by frequency.

Frequency	Right Ear	Left Ear
1 kHz	58 (50.9%)	50 (44.6%)
2 kHz	34 (29.8%)	35 (31.5%)
4 kHz	45 (39.5%)	43 (38.7%)

**Table 3:** Tympanogram and hearing screening: summary of alterations.

	N	%
U/L Tympanogram	104	9.7
U/L Hearing Screening	9	0.8
U/L Tympanogram + Hearing Screening	24	2.2
B/L Tympanogram	81	7.6
B/L Hearing Screening	3	0.3
B/L Tympanogram + Hearing Screening (U/L)	31	2.9
B/L Tympanogram + Hearing Screening (B/L)	51	4.8
B/L Hearing Screening + Tympanogram (U/L)	7	0.7

U/L—unilateral; B/L—bilateral.

**Table 4:** Referrals and interventions recommended by on-site ENT specialists.

	N	%
ENT Consultation	123	56.7
Monitoring	30	13.8
Medication	20	9.2
Discharge	19	8.8
Other Consultation(s)	16	7.4
Wax Removal	5	2.3
Speech Therapy	4	1.8
**Total**	**217**	**100.0**

N—number; %—percentage.

**Table 5:** Otoscopy and tympanogram results: comparative analysis.

Medical Otoscopy	Type A (N = 42)	Type C1 (N = 60)	Type C2 (N = 159)	Type B (N = 173)
Normal	36 (86.4%)	50 (83.3%)	85 (53.5%)	33 (19.1%)
Otitis Media with Effusion	2 (4.8%)	3 (5.0%)	13 (8.2%)	91 (52.6%)
Acute Otitis Media	1 (2.4%)	0 (0.0%)	3 (1.9%)	8 (4.6%)
Tympanic Depression	0 (0.0%)	3 (5.0%)	23 (14.5%)	11 (6.4%)
Cerumen	2 (4.8%)	2 (3.3%)	11 (6.9%)	22 (12.7%)
Tympanosclerosis	0 (0.0%)	0 (0.0%)	2 (1.3%)	0 (0.0%)
Tympanic Perforation	0 (0.0%)	0 (0.0%)	0 (0.0%)	1 (0.6%)
Eustachian Tube Dysfunction	1 (2.4%)	2 (3.3%)	19 (11.9%)	7 (4.0%)

N—number; %—percentage.

## Data Availability

The raw data supporting the conclusions of this article will be made available by the authors on request.
